# Tear-Derived Exosome Proteins Are Increased in Patients with Thyroid Eye Disease

**DOI:** 10.3390/ijms22031115

**Published:** 2021-01-23

**Authors:** Jeong-Sun Han, Sung Eun Kim, Jun-Qing Jin, Na Ri Park, Ji-Young Lee, Hong Lim Kim, Seong-Beom Lee, Suk-Woo Yang, Dong-Jun Lim

**Affiliations:** 1 Department of Internal Medicine, Division of Endocrinology and Metabolism, Seoul St. Mary Hospital, College of Medicine, The Catholic University of Korea, Seoul 06591, Korea; winehan@catholic.ac.kr (J.-S.H.); juncheong1991@catholic.ac.kr (J.-Q.J.); 2 Department of Ophthalmology and Visual Science, Seoul St. Mary Hospital, College of Medicine, The Catholic University of Korea, Seoul 06591, Korea; sungeuni85@naver.com (S.E.K.); happy_nal@hanmail.net (N.R.P.); 3 Department of Pathology, Institute of Hansen’s Disease, Seoul St. Mary Hospital, College of Medicine, The Catholic University of Korea, Seoul 06591, Korea; jy1012sh@hanmail.net (J.-Y.L.); sblee@catholic.ac.kr (S.-B.L.); 4 Integrative Research Support Center, Laboratory of Electron Microscope, College of Medicine, The Catholic University of Korea, Seoul 06591, Korea; wgwkim@catholic.ac.kr

**Keywords:** exosomes, extracellular vesicle, eear fluids, Graves’ ophthalmopathy, ehyroid-associated ophthalmopathy, eytokines

## Abstract

Exosomes contain proteins, lipids, RNA, and DNA that mediate intercellular signaling. Exosomes can contribute to the pathological processes of various diseases, although their roles in ocular diseases are unclear. We aimed to isolate exosomes from tear fluids (TF) of patients with Thyroid eye disease (TED) and analyze the exosomal proteins. TFs were collected from eight patients with TED and eight control subjects. The number of TF exosomes were measured using nanoparticle-tracking analysis. The expression of specific proteins in the purified exosome pellets were analyzed using a Proteome Profiler Array Kit. Cultured normal orbital fibroblasts were incubated with TF exosomes from patients with TED and control subjects, and changes in inflammatory cytokine levels were compared. TF exosomes from TED patients showed more exosomes than the control subjects. The expression levels of exosomal proteins vitamin D-binding (VDB) protein, C-reactive protein (CRP), chitinase 3-like 1 (CHI3L1), matrix metalloproteinase-9 (MMP-9), and vascular adhesion molecule-1 (VCAM-1) were significantly increased in patients with TED, compared to those of controls. Orbital fibroblasts exposed to TF exosomes from patients with TED showed significantly higher levels of interleukin (IL)-6, IL-8, and monocyte chemoattractant protein-1 (MCP-1) production than those treated with control TF exosomes. Specific proteins showed higher expression in exosomes from TED patients, implying that they may play keys roles in TED pathogenesis.

## 1. Introduction

Thyroid eye disease (TED) is the most common extrathyroidal manifestation of autoimmune thyroid disease [1,2]. TED is characterized by enlarged extraocular muscles and increased fatty and connective tissue, resulting in exophthalmos, restricted extraocular muscle movements, and, in severe cases, visual field loss due to corneal breakdown or compressive optic neuropathy [3,4]. Therefore, early diagnosis and recognition of initial surgical treatment is essential to preventing further progression of the TED.

Eckstein et al. reported that the activation of inflammatory cytokines and thyroid-stimulating hormone receptor (TSH-R) autoantibodies in tears are key components promoting ophthalmic disease, as well as the progression of TED [5]. Recently, proteomic analysis of specific proteins in tear fluids (TF) of patients with TED was undertaken to better characterize disease activity and to identify potential biomarkers [6,7]. Dysregulated cytokine levels were previously detected in TF and those of interleukin (IL)-1β, IL-6, IL-13, IL-17A, IL-18, tumor necrosis factor (TNF-α), and regulated upon activation, normal T cell expressed and secreted (RANTES) were increased in patients with TED [8], while IL-7 was increased in patients with inactive TED [9,10]. Therefore, specific information from TF in TED patients may help to investigate the mechanisms that trigger and maintain TED.

Exosomes are 30–150 nm-sized extracellular vesicles that are present in numerous body fluids, such as plasma, urine, synovial fluid, breast milk, saliva, and tears [11,12,13,14]. Exosomes mediate intra- and extra-cellular signaling, modulate immune reactions, and are modulators in diseases [15]. Cytokines can play biological roles as soluble free forms or in exosome-associated forms, either surface-bound or encapsulated [16]. Grigor’Eva et al. first reported that exosomes could be found in the TF of healthy subjects. In these studies, TF was determined to contain significantly large concentrations of exosomes, and these have been suggested to be mediators of the pathogenesis of ophthalmic diseases, which will further an understanding of such conditions [17]. Recent studies showed that tear-derived exosomes (from patients with primary Sjogren syndrome [18], primary open-angle glaucoma [19,20], and multiple sclerosis [21]) provide non-invasive diagnostic potential or disease-activity biomarkers. However, there have been no studies investigating or characterizing exosomal proteins and cytokines that have the potential to induce TED.

In this study, we herein aimed to isolate exosomes from TF of patients with TED and to investigate specific proteins or cytokines in exosomes that are differentially expressed relative to healthy control subjects.

## 2. Results

### 2.1. Characteristics of Patients with TED and Control Subjects

Tear fluids were obtained from eight consecutive patients with TED, with a mean age of 49 years (range 29–73 years) and eight unmatched controls, with a mean age of 66 years (range 58–79 years). The TED and control groups had the same sex ratio (F:M 6:2). All TED patients had been diagnosed with Graves’ disease and had been treated with methimazole. Among them, five patients previously received glucocorticoid therapy in the acute phase of the disease. The duration of TED ranged from 6 to 72 months. The mean value of thyroid-stimulating immunoglobulin (TSI) bioassay (normal; <140%) was 372% (range, 157–684) [22]. The clinical activity score (CAS) ranged from 1 to 4, and the no signs or symptoms, only signs, soft tissue, proptosis, extraocular muscle, cornea, sight loss (NOSPECS) score ranged from 1 to 6. The clinical characteristics of the TED patients are described in Table 1.

### 2.2. Characteristics of Exosomes Obtained from TF

**Figure 1:**
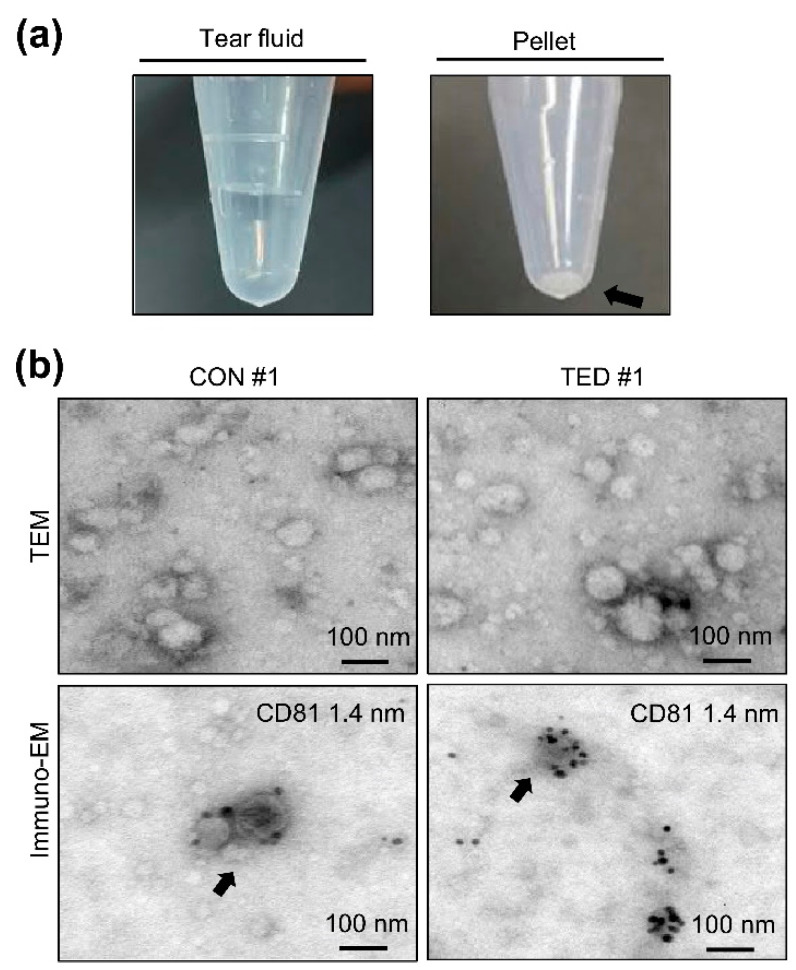
Isolation of exosomes from tear fluid. (**a**) Exosome pellets (black arrow) were obtained using the Exosome Plus^TM^ method for tear fluid. (**b**) Microscopic analysis of the morphology of isolated exosomes from control subjects and TED patients, based on TEM analysis. The upper panels show negative staining with unlabeled immuno-gold particles, and the lower panels show 1.4-nM immuno-gold labeling using an anti-CD81 antibody. Scale bar, 100 nm. CON—control subject; TED—thyroid eye disease; TEM—transmission electron microscopy; immune-EM—immuno electron microscopy.

**Figure 2:**
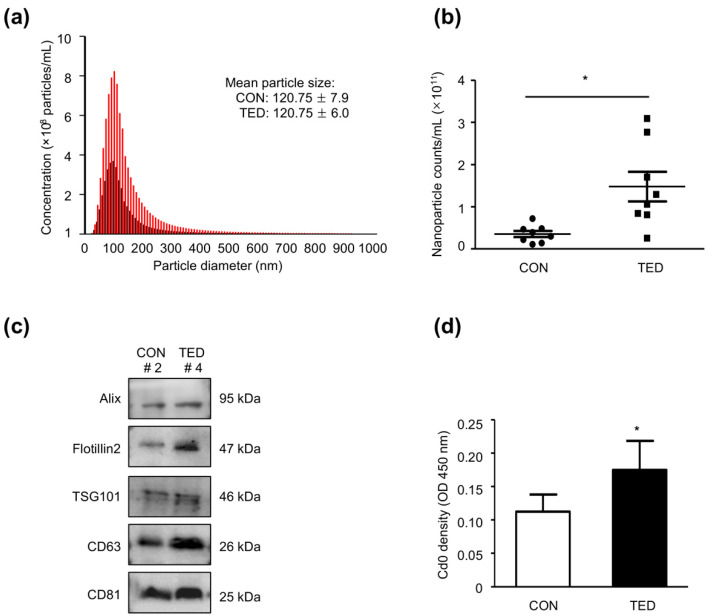
Tear-derived exosomes increased in patients with TED. (**a**) Nanoparticle tracking analysis of exosomes showing the exosome size distributions in the TED group (*n* = 8; red line) and the control group (*n* = 8; black line). Values are shown as mean ± SD. (**b**) The exosome concentrations increased dramatically in the TED group (*n* = 8; black squares) compared to that in the control group (*n* = 8; black circles). Statistical differences were determined by performing a paired *t*-test (* *p* < 0.05). (**c**) Western blot analysis of exosomes using antibodies against Alix, Flotillin 2, TSG 101, CD 63, and CD 81. The levels of exosome markers were higher in patients with TED than in control subjects. (**d**) Enzyme-linked immunosorbent assay (ELISA) analysis showed higher exosome-contained CD 9 levels in patients with TED (*n* = 5; black column) when compared with those of control subjects (*n* = 5; white column). Statistical differences were determined using a paired *t*-test (* *p* < 0.05). CON—control subject; SD—standard deviation; TED—patient with thyroid eye disease.

A representative image of an isolated exosome pellet obtained after centrifugation is shown in Figure 1a. Figure 1b shows the morphologies of exosomes as determined by Transmission electron microscopy (TEM) with immune-gold labeling of Cluster of Differentiation 81 (CD 81), a specific marker of exosomes. Exosome pellets from patients with TED and control subjects both contained microparticles with a round or ovoid shape that were CD 81-positive. Nanoparticle-tracking analysis (NTA) revealed no differences in the sizes of the exosomes, where the mean diameter was 120.75 ± 6.0 nm in the TED group and 120.75 ± 7.9 nm in the control group (Figure 2a). However, the mean exosome concentration was 2.3-fold higher (*p* < 0.05) in the TED group (1.23 × 10^11^ nanoparticle/mL) than in the control group (5.31 × 10^10^ nanoparticle/mL), as shown in Figure 2b. Antibodies against Alix, Flotillin 2, TSG 101, CD 63, and CD 81, which are the markers of exosomes, were readily detected by Western blot analysis in both groups. Interestingly, the levels of marker protein expression were remarkably higher in the TED group than in the control group (Figure 2c). We further characterized the exosomes by enzyme-linked immunosorbent assay (ELISA) with an anti-CD 9 antibody, finding that the optical density of CD 9 was significantly higher (*p* < 0.05) in exosomes from patients with TED than from control subjects (Figure 2d).

### 2.3. The Levels of Five Proteins Increased Significantly in Exosomes from TF of Patients with TED

**Figure 3:**
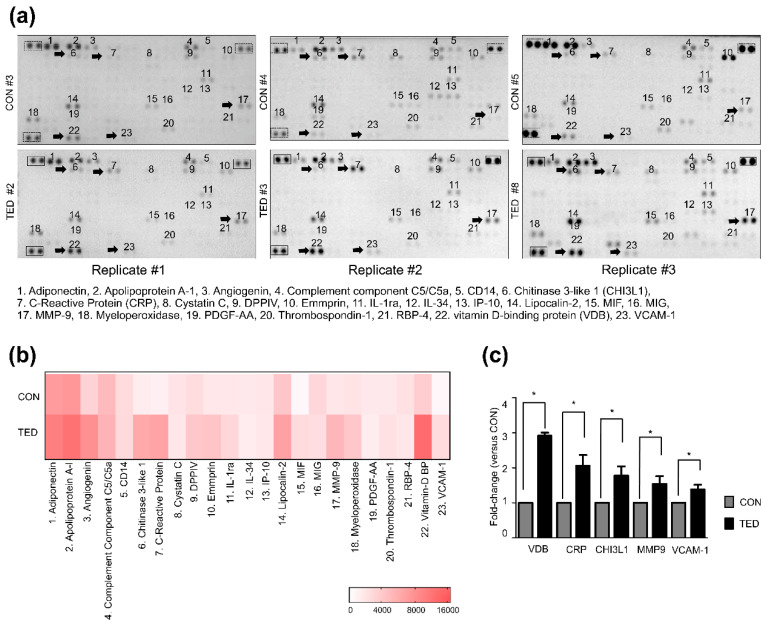
Analysis of exosome-contained proteins using the Proteome Profiler Human XL Cytokine Array. (**a**) Exosomes (100 μg) were analyzed using the Proteome Profiler Human XL Cytokine Array Kit. Representative images of array membranes corresponding to three different patients with TED and three control subjects. The numbered spots indicate proteins that showed different signal intensities among the array membranes. Five proteins showing the greatest differences are indicated with black arrows in each panel. (**b**) Heatmap analysis showing signal-density measurements of the data shown in (**a**). Of 105 human proteins targeted by the kit, 23 proteins were detected. The spots in the line boxes were used for signal normalization. The scale bar represents unitless measurements that were determined based on the pixel density. (**c**) The data shown represent at least 1.4-fold changes in the protein levels (normalized to array reference marked with the line boxes) in patients with TED versus control subjects. The mean pixel-density value of the control subject was set to 1.0, and the fold-changes were calculated for each protein. The data shown are presented as the mean fold-change ± SD (*n* = 3). Statistical differences were determined using a paired *t*-test (*p < 0.05). CON—control subject; SD—standard deviation; TED—patient with thyroid eye disease.

We used a Proteome Profiler Human XL Cytokine Array Kit to detect specific inflammation-related proteins and their expression levels in exosomes from tear fluids. Of the 105 human proteins contained in the kit, 23 proteins were detected (Figure 3a,b). Among them, five proteins, including vitamin D-binding (VDB) protein, C-reactive protein (CRP), chitinase 3-like 1 (CHI3L1), matrix metalloproteinase-9 (MMP-9), and vascular adhesion molecule-1 (VCAM-1) showed significantly increased expression levels (*p* < 0.05) in exosomes from TED patients compared to controls (2.9-, 2.1-, 1.7-, 1.5-, and 1.4-fold increases, respectively) (Figure 3c).

### 2.4. Levels of IL-6, IL-8, and MCP-1 Increased in Orbital Fibroblasts after Stimulation with Exosomes from Tear Fluids of Patients with TED

**Figure 4:**
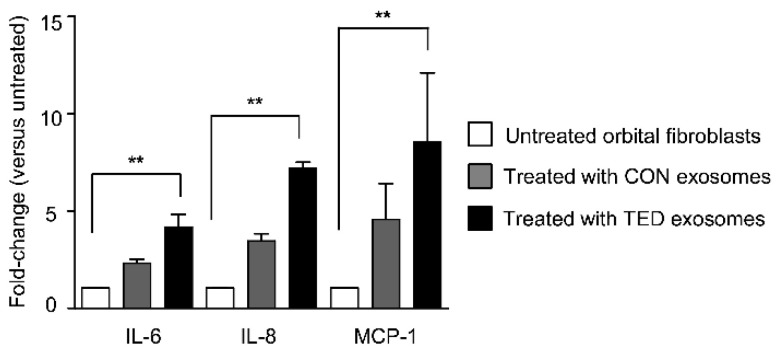
Tear-derived exosomes stimulate cytokine production on normal orbital fibroblasts. The concentrations of IL-6, IL-8, and MCP-1 were measured by ELISA analysis. The mean cytokine concentrations of untreated cells were set to 1.0, and the fold-changes were calculated. The data shown are presented as mean fold-change ± SD (*n* = 5). Statistical differences were determined using a paired *t*-test (** *p* < 0.01). CON—control subject; TED—patient with thyroid eye disease.

To determine the inflammatory effect of exosomes on orbital fibroblasts, healthy orbital fibroblasts were stimulated separately with exosomes from patients with TED and control subjects. Previously, numerous cytokines, such as IL-6, IL-8, IL-10, IL-16, TNF-α, RANTES, and MCP-1 were detected at higher levels in the orbital tissues of patients with TED, where they acted as potent stimulators of TED pathogenesis [23]. Especially, IL-6 and IL-8, which are mostly derived from orbital fibroblasts, have been known to be associated with the development of TED [24] and MCP-1, a chemoattractant that induces mononuclear infiltration, plays a role in promoting inflammation and recruiting macrophages [25]. Here, the levels of IL-6, IL-8, and monocyte chemoattractant protein-1 (MCP-1) were determined by in vitro ELISA analysis. Normal orbital fibroblasts were incubated with or without 5 µg exosomes for 24 h. Interestingly, the concentrations of IL-6, IL-8, and MCP-1 significantly increased (*p* < 0.05) in orbital fibroblasts exposed to exosomes from patients with TED, as compared to treatment with control exosomes. Specifically, the respective increases after treatment with patient or control exosomes were as follows: IL-6: 4.2-fold versus 2.3-fold; IL-8: 7.2-fold versus 3.5-fold; and MCP-1: 8.6-fold versus 4.6-fold (Figure 4).

## 3. Discussion

In this prospective and controlled study, we isolated exosomes and analyzed exosomal proteins using TF from patients with TED and healthy controls. Detection and parallel measurements of 23 exosomal proteins could be performed using a proteome cytokine array kit with high sensitivity. We demonstrated that in TF, the number of exosomes was 2.3-fold higher in patients with TED than in control subjects. The increased number of exosomes may lead to activation of their functions and the release of more inflammatory cytokines, which may promote tissue remodeling and inflammation. Taken together, the increase in the number of exosomes and expression of specific proteins in exosomes from tear fluid may reflect pathological processes occurring in TED.

Although inflammation occurs in the orbital space where activated T lymphocytes and macrophages release cytokines, inflammatory changes in the lacrimal glands also occur in TED. Evidence suggests that the TSH-R, the key target molecule of autoantibodies in TED, is expressed in the lacrimal glands [5]. Lymphocyte infiltration and interstitial edema have been found during pathological observations of the lacrimal glands, lacrimal gland enlargement has been seen in radiological studies, and lacrimal glands are reported to show increased production of pro-inflammatory cytokines, which may result in proteomic changes in tear fluid [26,27,28]. In addition, conjunctival invasion is easily observed in the early stages of TED before orbitopathy develops, and the pathological process is similar to the processes associated with extraocular muscle inflammation and lymphocyte infiltration in the posterior orbital space [29]. These data suggest that ocular surface tissues are indicative targets for autoantibodies in TED. As exosomes are released from almost all cells, the source of exosomes in tear fluid may include lacrimal gland cells, corneal and conjunctival fibroblasts, and epithelial cells [30].

In this study, we detected five proteins with higher expression levels in exosomes from patients with TED than in those from control subjects. MMPs are enzymes that play key roles in tissue remodeling during fibrotic and inflammatory processes. Their expression levels are very low in normal tissues, but can be increased by inflammatory cytokines, cell–cell interactions, hormones, and growth factors [31,32]. Previous reports showed that the serum concentrations of MMP-9 were elevated in TED patients and were associated with the CAS [33]. Moreover, exosomes or extracellular vesicles from cancers, inflammatory diseases, or even myocardial infarction carry MMP9 and are involved in extensive biologic process of effects as a key biomarker [34]. CRP and VCAM-1 are well-established biomarkers of systemic inflammation and exhibit increased expression during inflammation. VCAM-1 is increased in the serum of TED patients, and previous evidence suggests that under conditions such as high levels of inflammation and chronic diseases [35,36,37].

CHI3L1 is a glycoprotein that mediates inflammation, macrophage polarization, apoptosis, and carcinogenesis. CH13L1 promotes inflammatory cytokine production (including TNF-α, IL-1β, IL-6, and IFN-γ), connective tissue growth, fibrosis, and cancer proliferation [38,39]. In addition, one study showed that CHI3L1, one of the two most abundant exosomal proteins released by macrophages, can induce pancreatic ductal adenocarcinoma cellular resistance to gemcitabine through ERK (extracellular-signal-regulated kinase) activation [40], indicating that CHI3L1 might be a key player in cancers or inflammatory diseases. However, because this protein was not found in ExoCarta, (http://www.exocarta.org), a manually curated web-based compendium of exosomal proteins, further investigations of the biological roles of exosomal CHI3L1 protein might be considered as a future study.

VDB protein is a glycosylated alpha-globulin that activates vitamin D metabolites through transport processes. In addition to the role of transporting vitamin D metabolites, VDB protein also functions as a modulator of immune and inflammatory responses and activates macrophages, which could affect the immune system. VDB protein is widely distributed in various tissues and can be detected in plasma, cerebrospinal fluid, seminal fluid, saliva, and breast milk [41]. A previous study targeting pulmonary sarcoidosis showed that major proteins derived from bronchoalveolar lavage fluid (BALF) are different in their distribution or numbers from those within the exosome from BALF, which indicated that exosomal proteins including VDB protein might play unique roles in the pathogenesis of disease [42]. Although TED has no association with vitamin D-dysregulated diseases like pulmonary sarcoidosis, VDB was the most abundant exosomal protein in our study and might play a role as an immune mediator within the exosome, which should be confirmed in a future study.

In TF from patients with TED, increased levels of cytokines, such as IL-1β, IL-6, IL-13, IL-17A, IL-18, TNF-α, and RANTES were detected in previous studies [8,10]. However, in this study, even though antibodies against the above cytokines were included in the Proteome Profiler Human XL Cytokine Array Kit, we could not detect differences in their levels. Cytokines can exist as soluble free molecules or can be released in encapsulated form in exosomes, depending on the characteristics of a biological system. When cytokines are encapsulated in exosomes, they are not detectable using standard cytokine assays. Fitzgerald et al. reported that IL-6 and IL-13 were more often found in a free form, whereas IL-17 and TNF-α were found in greater levels in exosomes in most systems. Moreover, they found that exosome-associated cytokines can either be surface-bound or encapsulated, according to the characteristics of different systems. They also found that IL-18 and TNF-α were preferentially encapsulated in exosomes in the majority of systems and that IL-6 was mostly found encapsulated in exosomes in body fluids [16]. In this study, we tested the hypothesis that some cytokines may be encapsulated in exosomes, rendering increased levels in exosomes undetectable in cytokine assays.

Finally, we demonstrated that exosomes in TF triggered orbital fibroblasts to release the inflammatory cytokines, IL-6, IL-8, and MCP-1 in vitro. These findings suggest that not only the number of exosomes, but also the increased abundance of specific proteins in exosomes can activate inflammatory response by orbital fibroblasts, which are the targeted cells in TED. Increased MMP-9 and CH13L1 levels in exosomes, and extracellular matrix modulating and fibrosis-promoting exosomal activity may play especially important roles in TED, as orbital tissue remodeling and fibrosis are integral parts of the disease mechanism. Previous reports showed that nuclear Sp1 protein is more abundant in orbital fibroblasts, and its binding to specific sites on DNA is greater than regular fibroblasts to modulate cytokine response [43,44]. Moreover, SP1 was shown to be the main target of delivered miRs in leukemic [45] and other cancer models [46]. In the future, exosomal miRs may play a critical role in orbital fibroblast communications.

A limitation of this study is that we did not categorize patients with TED who might have also had dry eye disease. Not all, but many TED patients complain of dry eye symptoms, and many molecules, including MMP-9, have been reported to be dysregulated in dry eye disease [47]. Dry eye disease can result from increased exposure of the ocular surface due to a wider interpalpebral fissure but also from pathologic inflammation in the ocular surface due to TED. However, because the control group did not have ocular surface diseases, including dry eye disease, we believe that the findings in this study are meaningful for comparing the groups in association with exosomes. Another is the age mismatch between the TED patients and control subjects, which might induce a more exaggerated response in younger TED patients than older control subjects. However, even the older patients within the TED group also showed higher responses than the control subjects, indicating that the age might not affect the results. In conclusion, the number of exosomes and level of exosomal cytokines were higher in the tear fluids of patients with TED. Specific proteins, such as VDB and CHI3L1, showed higher expression in exosomes from TED patients, implying that they might play keys roles in TED pathogenesis.

## 4. Materials and Methods

### 4.1. Patients

We evaluated sixteen participants, including patients with TED (*n* = 8) and healthy control patients (*n* = 8). Consecutive patients with TED were enrolled in this study who were scheduled to undergo decompression surgery under general anesthesia. Patient history, including smoking (current, past, and never smokers), glucocorticoid therapy, and radiation therapy (yes/no) were also obtained. TED activity was evaluated by the following approaches: using a serum thyroid-stimulating hormone receptor (TSH-R) antibody (Roche Diagnostics, Mannheim, Germany), performing serum thyroid-stimulating immunoglobulin (TSI) bioassays (Diagnostic Hybrids, Inc., Athens, OH, USA), and determining clinical activity scores (CAS) and no signs or symptoms; only signs; soft tissue; proptosis; extraocular muscle; cornea; sight loss (NOSPECS) scores. The CAS represents the sum of seven scores related to the presence (1) or absence (0) of spontaneous retrobulbar pain, pain on attempted up or down gaze, swelling of the eyelids, redness of the eyelids, redness of the conjunctiva, conjunctival edema, and inflammation of the caruncle (CAS range: 1–7). The NOSPECS score was determined as follows: 0—no signs or symptoms; 1—only signs; 2—soft tissue involvement with symptoms and signs; 3—proptosis (≥20 mm); 4—extraocular muscle involvement; 5—corneal involvement; 6—sight loss (≤0.67) (NOSPECS score range: 0–6) [48]. Control subjects (patients with nasolacrimal duct obstruction) were also studied, and tear fluids were obtained during the dacryocystorhinostomy operation. Patients in the control group had no thyroid disease or other ocular diseases such as dry eye disease, conjunctivitis, meibomian gland dysfunction, and glaucoma, which may induce ocular surface changes.

### 4.2. Collection, Isolation, and Quantification of Exosomes from Tear Fluids

TFs were collected five times by gently and intermittently placing a polyvinyl acetal spears eye sponge (Beaver-Visitec International, Waltham, MA, USA) on the inferior conjunctival sac at the operating room under general anesthesia. The eye sponge was introduced into a 0.2-mL polymerase chain reaction (PCR) tube (Eppendorf, Hamburg, Germany), and the TF was obtained by centrifugation at 13,000 rpm at 4 °C for 30 min [49]. The samples were stored in new tube at −80 °C until further analysis. Exosomes were isolated using the Exosome Isolation Reagent (Exosome Plus, Seoul, Korea) [50]. The pellet was suspended in filtered Phosphate-buffered saline (PBS). Quantification of exosomes was performed using a bicinchoninic acid (BCA) assay (Thermo Fisher Scientific, Waltham, MA, USA). The plates were vortexed for 30 s, and the absorbance at 540 nm was read using a microplate reader (BioTek Instruments, Winooski, VT, USA) and compared with a control protein.

### 4.3. Culturing Orbiral Fibroblasts

Normal human orbital fibroblasts were obtained from Prof S.-B. L. (Catholic University, Korea) and cultured in Dulbecco’s modified Eagle’s medium (Thermo Fisher Scientific, Waltham, MA, USA) supplemented with 10% fetal bovine serum (FBS; Gibco BRL) at 37 °C in 5% CO_2_. Orbital fibroblasts (10,000 cells/well) were co-cultured with two types of exosomes (5 μg/well), each from patients with TED and control subjects, in a 24-well plate for 24 h. The supernatants were then collected and stored at –20 °C until the levels of IL-8, IL-6, and MCP-1 were analyzed by enzyme-linked immunosorbent assay (ELISA) (R&D Systems, Inc., Minneapolis, MN, USA).

### 4.4. Transmission Electron Microscopy (TEM) and Immune-Gold Labeling

For anti-CD81 immunolabeling, exosome samples were incubated in 4% paraformaldehyde at 37 °C for 1 h, and 10 μL of the fixed exosomes was spotted onto formvar-coated Nickel grids (300 mesh. The grids were then blocked in PBS (pH 7.4) containing 1% BSA (*w/v*) for 1 h. After a 1-h incubation at 37 °C, the grids were washed five times in PBS (10 min/wash step). The grids were floated on drops of 1.4-nm anti-mouse-nanogold conjugate (Nanoprobes, Inc., Yaphank, NY, USA) in PBS for 1 h. Silver enhancement was completed using the High-quality Silver Enhancement Kit (Nanoprobes, Inc., Yaphank, NY, USA) for 2 min. Then, negative staining in 1% aqueous uranyl acetate was quickly performed, and all samples were wicked dry and allowed to air dry. Pellets were examined using a TEM (JEM-1010; Tokyo, Japan) at 100 kV.

### 4.5. Nanoparticle-Tracking Analysis (NTA)

Exosomes were analyzed by performing NTA (Exosome Plus, Seoul, South Korea). Measurements were repeated 15 times at different sub-volume positions at 25 °C. Analysis was performed using ExoCope tracker software, version 1.005. The intensity threshold was set to a level of 20 (quadrature noise level in digital numbers/pixel), the minimum tracked particle size was set to 50 nm, and the minimum separable particle distance was set to 5.7 pixels. The system was calibrated with 100-nm polystyrene beads at five different concentrations. Each sample was diluted 1:500–1:2000 in 200 nm-filtered PBS, such that 30–200 particles were observed in each field of the image [51].

### 4.6. ELISA Analysis

The Exosome ELISA Complete Kit (System Biosciences, Palo Alto, CA, USA) was used to determine CD9 levels in exosomes. The presence of CD9 in exosomes was determined by measuring the absorbance at 450 nm with a microplate reader (BioTek, Winooski, VT, USA).

### 4.7. Western Blot Analysis

Exosome (10 μg) were electrophoresed via 4–15% SDS-PAGE and transferred to polyvinylidene difluoride (PVDF) membranes. After, blocking with 5% BSA, the PVDF membranes were incubated overnight at 4 °C with primary antibodies against ALG-2 interacting protein X (Alix), CD63 (Santa Cruz Biotechnology, Dallas, TX, USA), Flotillin 2, tumor susceptibility gene 101 (TSG101), and CD81 (Novus Biologicals, Littleton, CO, USA). Signals were generated using an Enhanced Chemiluminescence (ECL) Plus system (Amersham Biosciences, Piscataway, NJ, USA). All images were detected using a Syngene PXi 4 digital imager (Syngene, Frederick, MD, USA). Quantification of protein bands was performed using Fujifilm Multi Gauge software, version 3.0.

### 4.8. Proteome Profiler Human XL Cytokine Array Kit Analysis

Exosome (100 μg) purified from tear fluids of patients with TED or control subjects were analyzed using the Proteome Profiler Human XL Cytokine Array Kit (R&D Systems, Minneapolis, MN, USA), which contained 105 different capture antibodies that were spotted on a nitrocellulose membrane, according to the manufacturer’s protocol. Immuno-spots were imaged using a Syngene PXi 4 digital imager (Syngene, Frederick, MD, USA) and the data were analyzed using Fujifilm Multi Gauge software, version 3.0. For each analyte, the average signal of the triplicate line boxes was calculated, corrected for background signals, and normalized to the average signal of the membrane reference spots [52].

### 4.9. Statistical Analysis

Statistical analysis was performed using GraphPad Prism (GRAPHPAD Software Inc., San Diego, CA, USA). Results are expressed as the mean ± standard deviation (SD). All calculated statistical evaluations were performed using Student’s two-tailed *t*-test; ** *p* < 0.01 or * *p* < 0.05 was considered to reflect statistically significant differences.

## Figures and Tables

**Table 1:** Clinical Characteristics of Patients with Thyroid Eye Disease (TED), from whom Tear Fluids Were Obtained.

Characteristic	# 1	# 2	# 3	# 4	# 5	# 6	# 7	# 8
Age (years)	73	29	30	45	56	62	35	63
Sex	Female	Female	Male	Male	Female	Female	Female	Female
Smoking status *	No	No	Past	Past	No	No	No	No
Prior Glucocorticoid	Yes	Yes	Yes	Yes	No	No	No	Yes
Prior Radiation	No	No	No	No	No	No	No	No
TSI bioassay (%)	434	684	166	267	606	280	157	383
TSH receptor antibodies (IU/L)	16.99	>40	1.36	2.14	2.43	1.45	0.59	4.53
TED duration (mouths)	8	36	17	72	20	10	6	24
CAS	4	3	1	2	2	1	1	3
NOSPECS	6	5	3	4	4	6	1	5

CAS—clinical activity score; NOSPECS—no signs or symptoms, only signs, soft tissue, proptosis, extraocular muscle, cornea, sight loss; TSH—thyroid-stimulating hormone; TSI—thyroid-stimulating immunoglobulin; TED—thyroid eye disease; * Smoking status: “no” means never-smoked; “past” means ex-smoker.

## Data Availability

The data presented in this study are available from the corresponding authors [D.-J.L. and S.-W.Y.] on request.
